# Application of Artificial Neural Network for predicting biomass growth during domestic wastewater treatment through a biological process

**DOI:** 10.1002/elsc.202200058

**Published:** 2023-04-13

**Authors:** Mpho Muloiwa, Megersa Dinka, Stephen Nyende‐Byakika

**Affiliations:** ^1^ Department of Civil Engineering Tshwane University of Technology Pretoria South Africa; ^2^ Department of Civil Engineering Science University of Johannesburg Johannesburg South Africa

**Keywords:** biological treatment process, DO concentration, Artificial Neural Network, temperature, organic and inorganic matter, oxygen uptake rate

## Abstract

The biological treatment process is responsible for removing organic and inorganic matter in wastewater. This process relies heavily on microorganisms to successfully remove organic and inorganic matter. The aim of the study was to model biomass growth in the biological treatment process. Multilayer perceptron (MLP) Artificial Neural Network (ANN) algorithm was used to model biomass growth. Three metrics: coefficient of determination (*R* ^2^), root mean squared error (RMSE), and mean squared error (MSE) were used to evaluate the performance of the model. Sensitivity analysis was applied to confirm variables that have a strong influence on biomass growth. The results of the study showed that MLP ANN algorithm was able to model biomass growth successfully. *R* ^2^ values were 0.844, 0.853, and 0.823 during training, validation, and testing phases, respectively. RMSE values were 0.7476, 1.1641, and 0.7798 during training, validation, and testing phases respectively. MSE values were 0.5589, 1.3551, and 0.6081 during training, validation, and testing phases, respectively. Sensitivity analysis results showed that temperature (47.2%) and dissolved oxygen (DO) concentration (40.2%) were the biggest drivers of biomass growth. Aeration period (4.3%), chemical oxygen demand (COD) concentration (3.2%), and oxygen uptake rate (OUR) (5.1%) contributed minimally. The biomass growth model can be applied at different wastewater treatment plants by different plant managers/operators in order to achieve optimum biomass growth. The optimum biomass growth will improve the removal of organic and inorganic matter in the biological treatment process.

**Abbreviations**
ANN: Artificial Neural Network
$CO_{2}$
: carbon dioxide
COD: chemical oxygen demand
DO: dissolved oxygen
EC: electrical conductivity
g/L: grams per liter
g/m^3^
: grams per cubic meter
$H_{2}O$
: water
L/min: liters per minute
*O*_2_
: oxygen
$PO_{4}$
: phosphate
MATLAB: matrix laboratory
mg/L: milligrams per liter
MLP: multilayer perceptron
MSE: mean squared error
$NH_{3}$
: ammonia
OD: optical density
ORP: oxidation reduction potential
OUR: oxygen uptake rate
pH: power of hydrogen
*R* ^2^
: coefficient of determination
RMSE: root mean squared error
SSE
: sum of squared error
SST
: total sum of squares
TDS: total dissolved solids
WWTP: wastewater treatment plant

## 1 INTRODUCTION

Domestic wastewater is generated from activities such as food preparation, toilet flushing, and bathing [1]. Wastewater treatment is achieved through a number of treatment processes: physical, biological, and chemical [2]. The physical treatment process is responsible for removing large particles using physical techniques such as screens, grit chambers, and sedimentation tanks [3]. The objective of the biological treatment process is to decompose organic matter and oxidize inorganic matter present in wastewater [4]. The chemical treatment process is usually the final step responsible for chemical stabilization and disinfection of wastewater before it is discharged into the environment [5].

In the biological treatment process, the removal of organic and inorganic matter is achieved using microorganisms [6, 7]. When microorganisms decompose organic and oxidize inorganic matter, they multiple and increase in population, which result in an abundance of microorganisms that are essential in the biological treatment process. The chemical reaction between microorganisms, organic and inorganic matter is shown in Equation (1), defined by ref. [8].

(1) $$\begin{array}{ccc} & & organic\;matter+O_{2}+NH_{3}\\ & & \;+PO_{4}^{-3}\overset{microorganisms}{\to }new\;cells+CO_{2}+H_{2}O \end{array}$$

When microorganisms consume organic and inorganic matter present in wastewater, the resultant is carbon dioxide, water, and new cells. New cells represent an increase in biomass, which is defined as the mass of microorganisms in the biological treatment process [9]. In order to have a successful biological treatment process, continuous growth of microorganisms need to be maintained in the biological treatment process. If growth of microorganisms’ does not take place, slow removal of organic and inorganic matter will take place and the reaction will not be satisfied.

**PRACTICAL APPLICATION**

- Temperature contributes 47.2% toward biomass growth.
- DO concentration contributes 40.2% toward biomass growth.
- Three growth phases (lag, exponential, stationary phases) were observed in the biological treatment process.
- Oxygen uptake rate (0.623**) showed a positive relation toward biomass growth.
- Aeration period (0.603**) showed a positive relation toward biomass growth.

The challenge experienced in the biological treatment process is that growth of microorganisms is slow, which decelerates the removal of organic and inorganic matter. Microorganisms’ growth is defined in four phases during the consumption of organic and inorganic matter: lag, exponential, stationary, and death phase. In the lag phase, microorganisms take time to acclimatize to their new environment in the biological treatment process. While the acclimatizing process takes place, there is no growth of microorganisms, which implies that there is no consuming of organic and inorganic matter in the biological treatment process [10].

**FIGURE 1:**
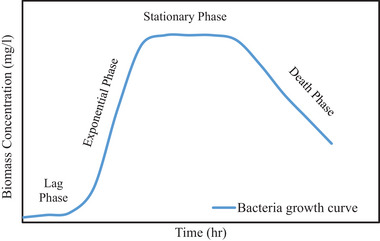
Bacteria growth rate curve (Metcalf and Eddy, 2003).

The exponential growth phase is the most rapid growth possible. In this phase, microorganisms present in the biological treatment process double at a high rate [11]. The stationary phase defines the no growth phase. Although there is no growth in the stationary phase, microorganisms continue to multiply and produce additional biomass while some continue to die [12, 13]. The stationary phase occurs due to the depletion of organic and inorganic matter, and the presence of toxic substrate in the biological treatment process. The last phase is the death phase. In this phase, nutrients required for biomass growth are completely used up, which indicates successful removal of organic and inorganic matter [14]. The four phases are defined in Figure 1.

In order to improve microorganisms’ growth, plant managers/operators resort to high airflow rates that increase DO concentration in the biological treatment process. The increase in DO concentration will guarantee abundance of oxygen for respiration and survival of microorganisms. A study conducted by Hasan et al. [15] reported that DO concentrations of 5.26 and 2.94 mg/L were measured at airflow rates of 2 and 0.3 L/min, respectively. This shows that increasing airflow rate will increase DO concentration in the biological treatment process. This is because high airflow rates produce copious quantities of bubbles, covering the entire surface area of the biological aeration unit. Bubbles carry air containing oxygen; therefore, an increase in the number of air bubbles increases the DO concentration [16, 17].

In addition, high airflow rates have been reported to improve volumetric mass transfer coefficient (K_L_a) which is an important parameter in the biological treatment process. An increase in K_L_a will ensure that oxygen is transferred to microorganisms at a rapid rate. A study reported by Kan et al. [18] showed that airflow rates of 2.5 and 7 L/min produced K_L_a of 0.2 and 0.727 kg O_2_/h, respectively. This indicates that an increase in airflow rate will improve K_L_a. This is because high airflow rates increase the air velocity distribution which increases the shear force on the surface, resulting in an increase in K_L_a [19]. The increase in the shear force enables the airflow to penetrate the wastewater film rapidly compared with low airflow rates.

Although high airflow rates improve DO concentration and K_L_a, an increase in airflow does not significantly benefit the growth of microorganisms in the biological treatment process. A study reported by Belli et al. [20] showed that microorganisms’ growth of 5.74 and 5.17 g/L were measured at airflow rates of 6.4 and 1.6 L/min, respectively. The difference in microorganisms’ growth was 9.93%, which was minimal, yet the difference in airflow rate was 75%. High airflow rates produce large bubbles that have high buoyancy force which splits and destroys the microorganisms resulting in a slow grow and possible death of microorganisms. Therefore, high airflow rates will only result in high energy consumption in the biological treatment process which is a global concern [20, 21].

In addition, an increase in airflow rate does not significantly benefit the removal of organic and inorganic matter. A study conducted by Fajri et al. [22] reported that airflow rates of 34 and 59 L/min produced COD removal of 71.9% and 73.7%, respectively. A difference of 1.8% in COD removal was achieved in the expense of 42.4% airflow rate increase. Similar results were reported by Ata et al. [23], the author showed that airflow rates of 3 and 7.5 L/min produced ammonia removal of 36.5% and 38.5%. A difference of 2% in ammonia removal was achieved in the expense of 60% increase in airflow rate. Increasing airflow rate will only increase energy consumption without significant improvement in organic and inorganic matter removal therefore, an alternative approach is required.

The current research proposes an increase in temperature of wastewater rather than increasing airflow rates that consume high levels of energy in the biological treatment process. High wastewater temperature improves microorganisms’ growth and enhance COD and ammonia removal in the biological treatment process [24]. For example, temperatures of 30°C and 3°C produced microorganisms’ growth of 20 and 8 g/L, respectively [25]. This is because, at low temperatures, there is an observed stiffening of the membrane's lipids, causing a decreased efficiency of protein embedded in the membrane, hence a slow growth [26]. In addition, at low temperatures the chemical reaction of enzymes is slow, causing all activities to halt.

Similarly, high temperatures enhance COD and ammonia removal in the biological treatment process. A temperature of 35°C produced COD and ammonia removal of 157 and 15.9 mg/L whereas a temperature of 15°C produced COD and ammonia removal of 99 and 5.5 mg/L, respectively [27]. That is a difference of 36.9% (COD) and 65.4% (ammonia) removal between a temperature of 35°C and 15°C. This was because the growth and metabolic rate of microorganisms is high at high temperatures. When there is high population of microorganisms in the biological treatment process, the probability of decomposing organic matter and oxidizing inorganic matter increases. Furthermore, the interaction between organic matter, surface carbon, oxygen, and silicon enhance removal at high temperatures [28].

High wastewater temperatures improve the growth and metabolic rate of microorganisms, which in return will enhance the removal of COD and ammonia in the biological treatment process. Therefore, when modeling biomass growth, temperature of wastewater should be included as an input parameter. Hence, the aim of the current study is to model biomass growth in the biological treatment process. MATLAB programming software will be used to apply Artificial Neural Network (ANN) algorithm. ANN algorithm will produce a robust model that can be used to predict biomass growth in the biological treatment process. The model will assist plant managers/operators to maximize the growth of microorganisms and reduce the labor of manually measuring biomass growth in the biological treatment process.

## 2 MATERIALS AND METHODS

### 2.1 Research conceptual framework

**FIGURE 2:**
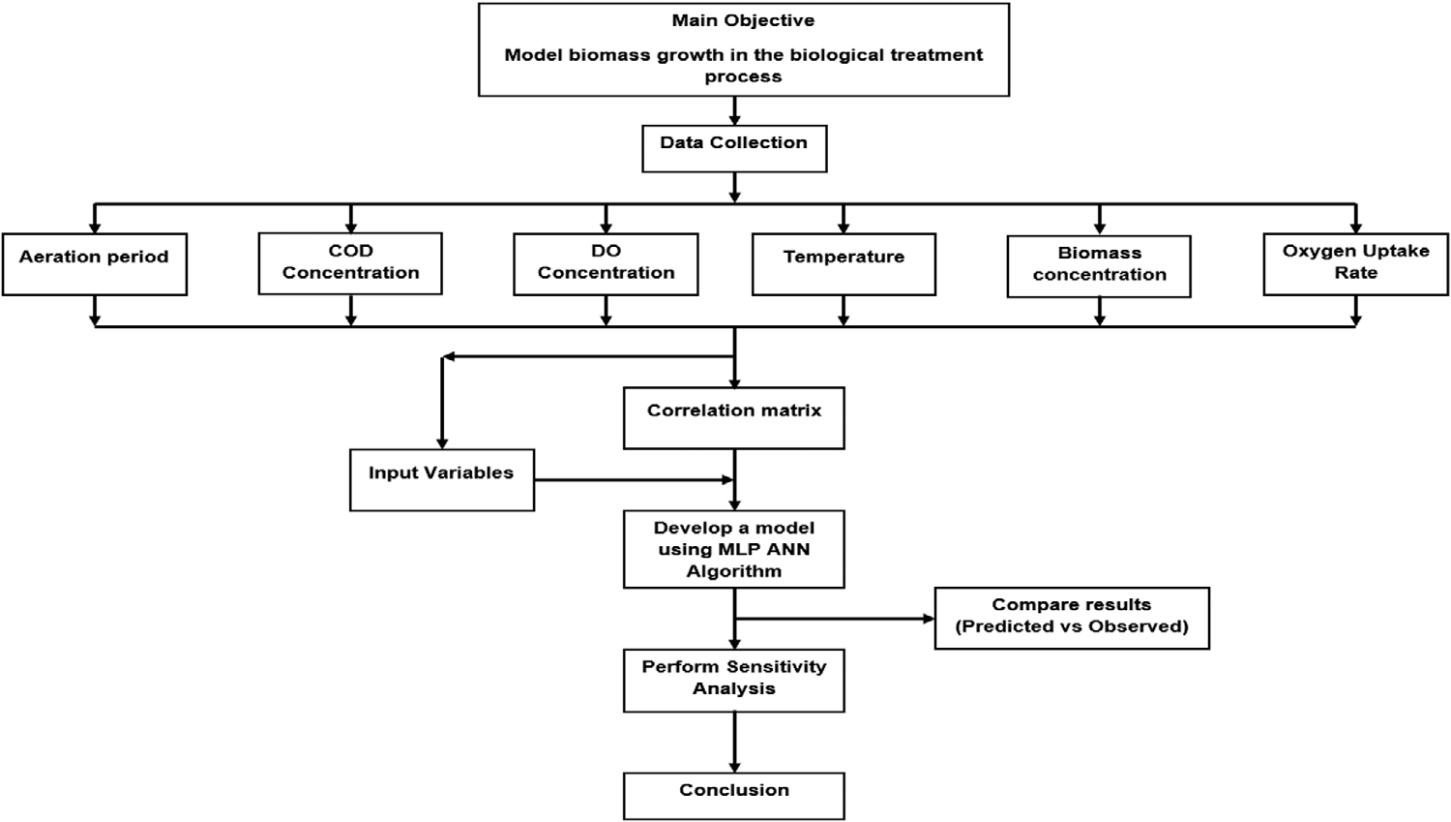
Research methodology conceptual framework.

Figure 2 presents the research methodology conceptual framework. The conceptual framework will be followed in order to achieve the aim of the current study.

### 2.2 Study area

**FIGURE 3:**
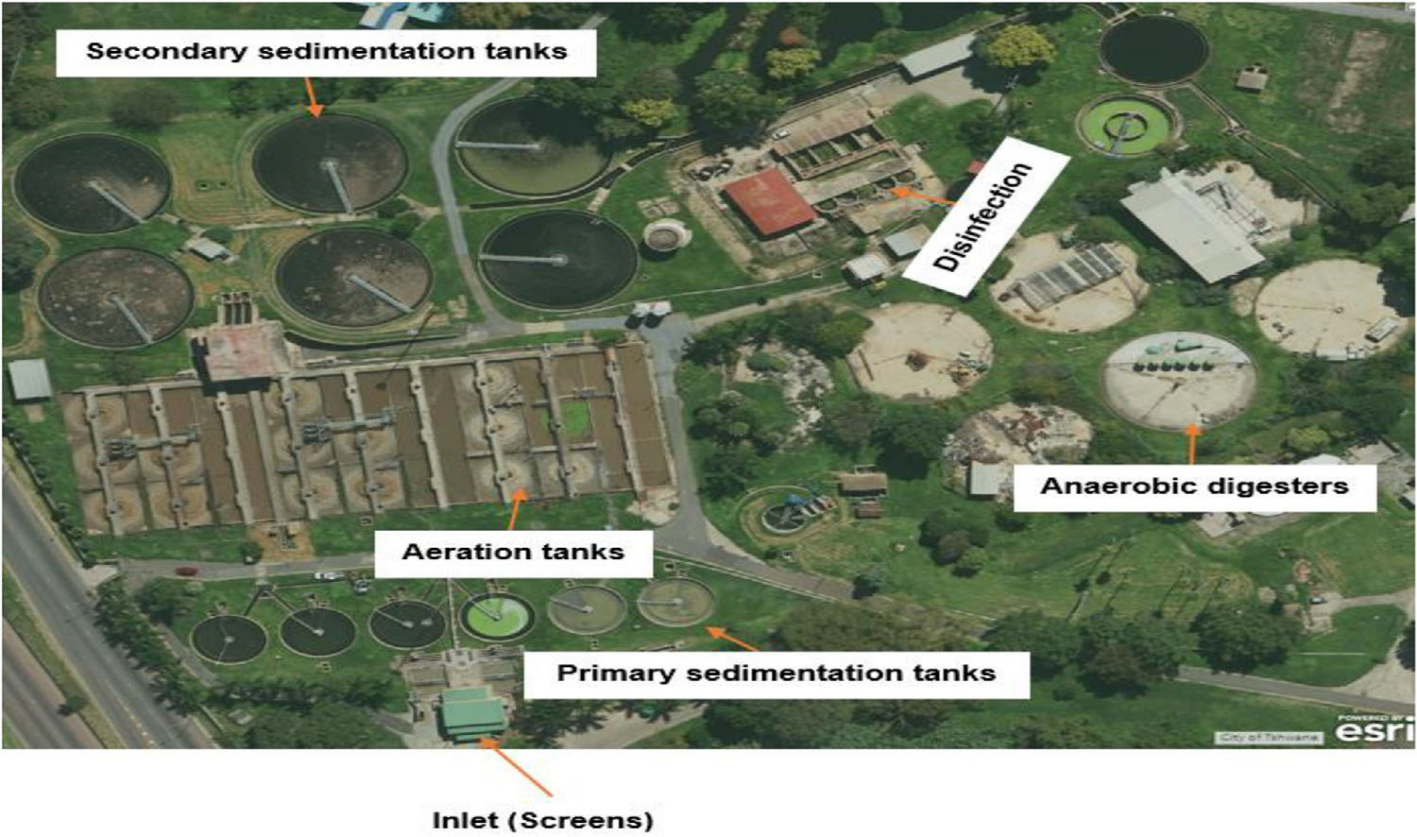
Daspoort wastewater treatment plant.

Daspoort WWTP was used as a case study in this research. The Daspoort WWTP is located in Pretoria, Gauteng Province, South Africa, coordinates (−25.733857, 28.177894). The Daspoort WWTP was designed and built between the years 1913 and 1920 to purify nine mega liters per day (ML/d) of wastewater. Over the years (1945–1947, 1973–1976, and 2009) the Daspoort WWTP has been upgraded and currently, the Daspoort WWTP has a total operating capacity of 55 ML/d. The Daspoort WWTP consists of three bioreactors, which are operated using surface aerators. Wastewater samples were collected from bioreactor number two because it was accessible easily compared to other bioreactors. The description of the Daspoort WWTP is shown in Figure 3.

### 2.3 Laboratory experiment setup

**FIGURE 4:**
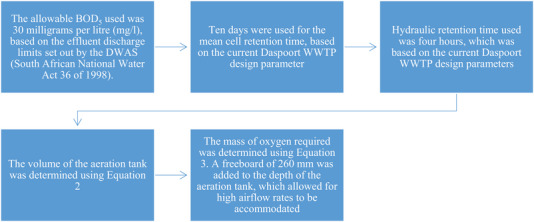
Biological aeration unit design flowchart.

The laboratory experiment mimicked the behavior of the Daspoort WWTP biological treatment process. Five factors were considered in designing the biological aeration unit as shown in Figure 4 [8, 29, 30].

**FIGURE 5:**
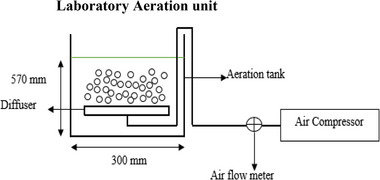
Laboratory aeration unit.

(2) V=θ×Q

(3) $$MO_{2}=\;Q\left(S_{o}-S\right)\left(\frac{10^{-3}kg}{g}\right)-1.42\left(P_{x}\right)$$

where *Q* is the volume of wastewater to be treated per day and θ is the hydraulic retention time. $MO_{2}$ is the mass of oxygen required, *Q* the volume of wastewater to be treated per day, $S_{o}$ and *S* are influent BOD/COD from the primary sedimentation tank and effluent BOD/COD from the biological aeration unit respectively, and $P_{x}$ is the waste activated sludge produced. The biological treatment process components and the schematic diagram are detailed in Table 1 and Figure 5, respectively.

**TABLE 1:** Components of the aeration unit.

Components	Type	Remarks
Aeration tank	Circular aeration tank. (Acrylic Material)	10–15 mm thick Acrylic material. Dimensions 570 × 300 mm. Total volume is 40 L. Working volume is 30 L
Dissolved oxygen meter and probe	Hanna HI98196 multi‐parameter water proof meter	Measures DO, pH, and ORP
Thermostat	Local manufacturer	Controls temperature in the range 0–40°C
Air pump	Waterfall Resun LP100	Can supply air between 0 and 140 L/min
Airflow meter	MF5712 200 L/min digital gas air nitrogen oxygen mass flow meter	Can measure airflow rate between 0 and 200 L/min
Air stone disc bubble diffuser	Growneer micro pore bubble diffuser	Diffuser is 20 cm in diameter
Digital wattmeter	Geewiz (Kill a watt)	Measures power between 0 and 3600 W

### 2.4 Wastewater collection

Wastewater was collected at the Daspoort WWTP, Pretoria, Gauteng Province, South Africa. The American Public Health Association method was used for the collection of wastewater. Non‐sterile powder free nitrile gloves were used to handle all equipment. Twenty‐five liters jerry can was used to collect the raw wastewater. An additional 2 L of activated sludge was collected from the return activated sludge. Wastewater samples were collected and aerated within 8 h after collection. Laboratory data was analyzed using descriptive statistics and correlation matrix.

### 2.5 Wastewater condition

Table 2 presents the average wastewater field conditions measured during the collection of wastewater at the Daspoort WWTP. Four parameters (pH, DO, oxidation reduction potential, and temperature) were measured using the Hanna HI98196 multi‐parameter water proof meter.

**TABLE 2:** Wastewater field condition

Parameters	Average concentration
pH	8.10–8.5
DO concentration	2.58–3.11 mg/L
Oxidation Reduction Potential	58.56–61.1 mV
Temperature	19.2–21.5°C

### 2.6 Wastewater characteristic analysis

Four wastewater characteristics were measured: COD, DO concentration, biomass concentration, and optical density (OD). The Standard Methods for the Examination of water and wastewater was used to analyze wastewater characteristics [31].

- COD was analyzed using closed reflux colorimetric (APHA method 5220). Spectrophotometer, Digestion vessels, Block heater, Microburet, and Ampule sealer, and DR3900 were the equipment used for the analysis.
- DO concentration was measured using the Hanna HI98194 Multi‐parameter pH/ORP/EC/TDS/Salinity/DO/Pressure/Temperature Waterproof Meter.
- Biomass concentration was measured using an evaporating dish, porcelain, silica glass, sieve, muffle furnace, steam bath, desiccator, drying oven, scale, magnetic stirrer, wide‐bore pipets, graduated cylinder, and beakers.
- OD was measured using a Spectrophotometer DR3900.

### 2.7 Disposal

After the completion of the aeration process, wastewater was disposed by flushing at the laboratory toilet. The disposal method allowed the wastewater to go back to the WWTP.

### 2.8 Aeration process

Aeration process was conducted twice a day (two samples collected). Two wastewater samples were aerated per day and each sample was aerated for 4 h (hydraulic retention time at Daspoort WWTP). This means that two different temperatures (35°C and 32.5°C) were applied during the aeration process at one of the airflow rates (5 L/min) per day. The same process was repeated the following day applying two different temperatures (30°C and 27.5°C) and the same airflow rate (5 L/min). The process was then repeated on all temperatures at different airflow rates. A thermostat (Table 1) was used to increase (heat up) the temperature of wastewater from field temperature (18–22.5°C) to the desired operating temperature. Ice cubes were used to decrease (reduce) the temperature of wastewater from field temperature to the desired operating temperature. Three samples were collected and analyzed during each aeration process:

- Initial concentration immediately after collection or before aeration process.
- Two‐hour concentration.
- Four‐hour concentration.

### 2.9 Oxygen uptake rate

**FIGURE 6:**
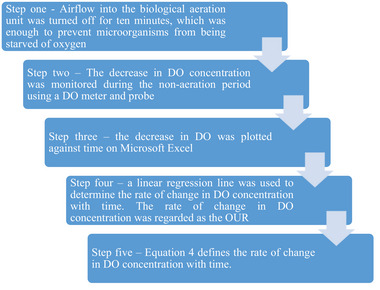
Determination of OUR flowchart.

The dynamic method described by Garcia‐Ochoa and Gomez [32] was used to determine the OUR. Five steps were followed to determine OUR as shown in Figure 6.

(4) $$\frac{dC}{dt}=\;-q_{O2}C_{X}$$

### 2.10 Application of Artificial Neural Network

**FIGURE 7:**
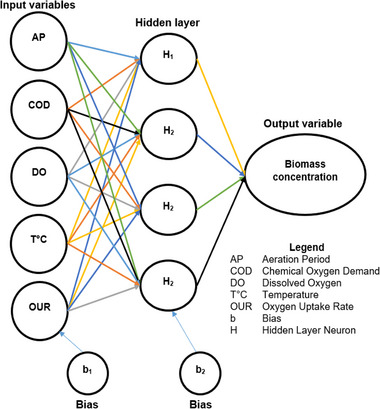
MLP ANN biomass concentration architecture.

A regression model was developed using MLP ANN algorithm on MATLAB programming software. Continuous data collected from laboratory experiments was used to develop the biomass growth model. The MLP ANN architecture is shown in Figure 7.

The general form of the MLP ANN algorithm is shown in Equation (5). Where *X* is the jth nodal value for the previous layer. *Y* is the ith nodal value in the current layer. *f* is the activation function. $W_{ij}$ is the weighting factor, and *b* is the bias of the ith node.

(5) $$Y_{i}=\;f\left(\sum_{j\;=\;1}^{N}W_{ij}X_{j}+bi\right)$$

**FIGURE 8:**
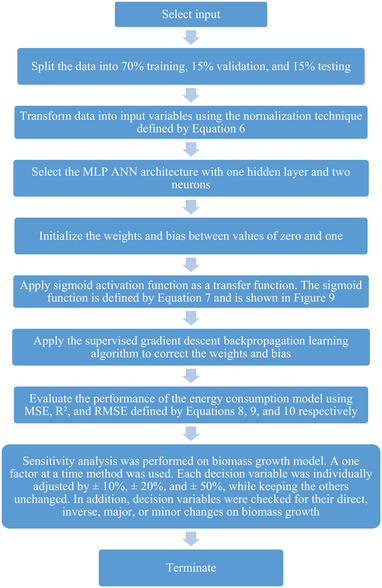
Application of MLP ANN algorithm flowchart.

**FIGURE 9:**
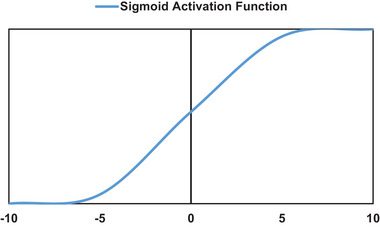
Sigmoid function [93].

Nine steps were followed to model biomass growth using MLP ANN algorithm as shown in Figures 8 and 9.

Where x(scaled) is the scaled sample data point, *x* is the sample data point, $min_{x}$ is the minimum value in the training dataset, $max_{x}$ is the maximum value on the training dataset.

(6) $$x\;\left(scaled\right)=\;\frac{x-min_{x}}{max_{x}-min_{x}}$$

(7) $$f\;=\frac{1}{1+e^{-x}}\;$$

(8) $$MSE=\sum_{i=1}^{n}\frac{\left({\hat{y}}_{i}-y_{i}\right){}^{2}}{N}$$

(9) $$R^{2}=\frac{1}{m}\sum_{j\;=\;1}^{m}\left(1-\frac{SSE}{SST}\right)$$

(10) $$RMSE=\sqrt{\frac{\sum_{i=1}^{n}\left({\hat{y}}_{i}-y_{i}\right){}^{2}}{N}}$$

where MSE is the mean squared error, ${\hat{y}}_{i}$ is the predicted value, $y_{i}$ is the observed value, *N* is the number of data points, *R* ^2^ is the coefficient of determination, SSE is the sum of squared error, and SST is the total sum of squares.

## 3 RESULTS AND DISCUSSION

### 3.1 Laboratory results

The descriptive statistics of the laboratory experimental results are presented in Table 3. The maximum and minimum measured biomass concentrations were 6.512 and 0.004 g/L in the biological treatment process, which was a difference of 99.9%. This indicates that biomass growth occurred because of the availability of nutrients, DO concentration, and an increase in wastewater temperature in the biological treatment process [6, 33]. Similarly, the maximum (10 mg/L) and minimum (0.52 mg/L) DO concentration showed a difference of 94.8%. This was due to aeration in the biological treatment process, which result in an increase in DO concentration [34, 35, 36].

**TABLE 3:** Descriptive statistic of the laboratory experimental data

	Biomass concentration (mg/L)	Aeration period (h)	COD (mg/L)	DO (mg/L)	Temperature (°C)	OUR (mg/L)
**Min**	0.004	0.000	31.00	0.520	15.000	0.020
**Max**	6.512	4.000	313.000	10.000	35.000	0.608
**Mean**	1.480	2.000	84.526	4.219	25.000	0.226
**SD**	1.497	1.633	53.700	1.773	6.105	0.115

The maximum OUR was 0.608 mg/L, which was due to the abundance of microorganisms in the biological treatment process compared with the minimum OUR of 0.020 mg/L. When microorganisms increase in population, OUR will increase because high number of microorganisms are consuming oxygen in the biological treatment process [36]. The maximum measured COD concentration was 313 mg/L, which indicates high organic concentration in wastewater. High organic matter will require advanced treatment technologies and extended aeration, which consume high levels of energy due to additional airflow supply required [37, 38].

### 3.2 Correlation matrix

**FIGURE 10:**
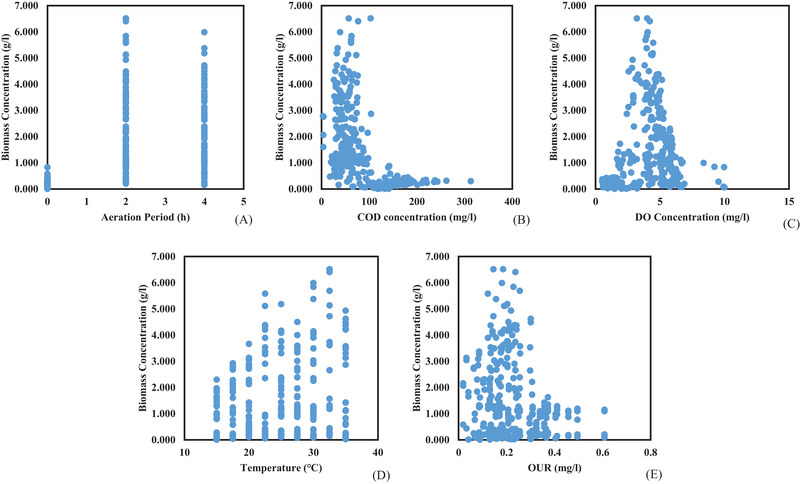
Relationship between biomass concentration and input variables.

Table 4 and Figure 10 present results of the correlation matrix and the graphical relationship between biomass concentration and input variables. Aeration period (0.603**), temperature (0.403**), and OUR (0.623**) had a positive relationship with biomass concentration. During the aeration process, microorganisms multiple due to consumption of nutrients, hence an increase in biomass takes place. Figure 10A shows that biomass growth was highest at the 2‐h mark which suggests that the stationary and death phases took place between the 2‐ and 4‐h mark. This indicates that when high wastewater temperatures are utilized, 4‐h hydraulic retention time is not necessary since the nutrients are already depleted and microorganisms have already reached the stationary and death phases [37, 38].

**TABLE 4:** Correlation matrix between biomass concentration and input variables

Correlations							
		BC	AP	COD	DO	T	OUR
**BC**	**PC**	1					
**AP**	**PC**	0.603**	1				
**COD**	**PC**	–0.578**	–0.860**	1			
**DO**	**PC**	–0.222**	0.135**	–0.250**	1		
**T**	**PC**	0.403**	0.000	0.123**	–0.848**	1	
**OUR**	**PC**	0.623**	0.802**	–0.695**	–0.101**	0.271**	1

* Correlation is significant at the 0.05 level (2‐tailed).

** Correlation is significant at the 0.01 level (2‐tailed).

Due to high wastewater temperatures, the lag and exponential phases were reached quicker as shown in Figure 10A. Temperature has been reported to provide a conducive environment that allows growth of microorganisms, hence temperature had a positive relation to biomass concentration in the biological treatment process [39, 40, 41, 42]. The highest biomass concentration (6.512 g/L) was measured at a temperature of 32.5°C whereas at normal wastewater temperature (18–22°C), the biomass concentration was measured to be 2.678–3.662 g/L as shown in Figure 10D. This is a clear indication that increasing wastewater temperature will result in rapid biomass growth. In essence, increasing wastewater temperature improves the lag and exponential phases in the biological treatment process.

OUR (0.623**) increased with an increase in biomass concentration in the biological treatment process. When OUR increases, it implies that microorganisms are alive and growth is taking place but when OUR decreases, it indicates that nutrients have been used up in the biological treatment process [43, 44]. Therefore, in order to maintain high OUR, nutrients should be available for microorganisms to continue feeding and reproducing. Hence, OUR has been used to indicate successful removal of organic and inorganic matter [45]. The relationship between biomass concentration and OUR is shown in Figure 10E. As shown in Figure 10E, highest biomass concentration was measured at average OUR of 0.25 mg/L, which indicates that there was even distribution of DO concentration and nutrients in the biological treatment process.

COD (−0.578**) had a negative relationship with biomass concentration. COD represents the nutrients that microorganisms feed on, hence when COD decreases, microorganisms increase in the biological treatment process [46]. This can also be justified by the relationship between COD and OUR which produced a negative relation of −0.695**. This relationship indicates that OUR can be used to monitor COD removal, since OUR shows that microorganisms are consuming oxygen in the biological treatment process [47, 48]. The relationship between biomass concentration and COD is shown in Figure 10B. In Figure 10B, the highest biomass (6.512 g/L) was measured at medium COD concentration (103 mg/L). This is because nutrients were still available in the biological treatment process, hence biomass growth was observed.

DO concentration (−0.222**) had a negative relationship with biomass concentration. For microorganisms to grow they require oxygen for respiration and survival, hence DO decreases when biomass concentration increases in the biological treatment process [49]. Similar to COD concentration, highest biomass concentration (6.512 g/L) was measured at medium DO concentration (4.02 mg/L) as shown in Figure 10C. This suggests that although microorganisms consumed DO, there was still abundance of DO concentration in the biological treatment process. This was due to the continuous airflow supply in the biological treatment process [50].

### 3.3 Modeling results

#### 3.3.1 MLP ANN model prediction accuracy

**FIGURE 11:**
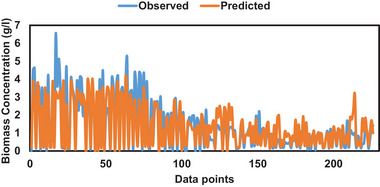
Observed versus predicted data during training phase.

**FIGURE 12:**
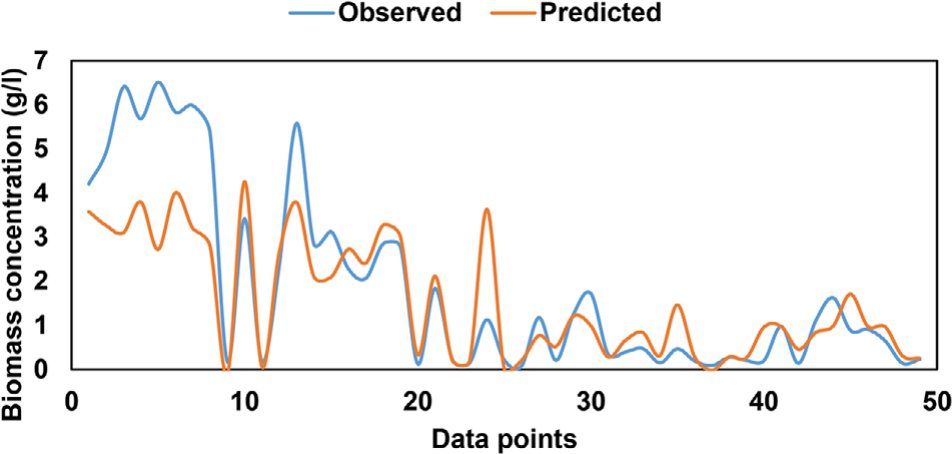
Observed versus Predicted data during validation phase.

**FIGURE 13:**
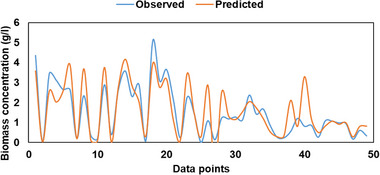
Observed versus predicted data during testing phase.

The prediction accuracy of the observed data points during training, validation, and testing phases are presented in Figures 11, 12, 13, respectively. During the training phase of the model, it can be observed that the model was able to predict the observed data points accurately. However, some of the peak data points between 0 and 75 were not predicted accurately as shown in Figure 11.

Similar observations can be seen during the validation phase of the model. The model struggled to predict peak values between data points 0 and 13 as shown in Figure 12. However, during the testing phase of the model, the model predicted the minimum, middle, and maximum data points accurately compared with the training and validation phases as shown in Figure 13.

Table 5 presents observed and predicted ANOVA test results. F‐statistic values were lower than the F‐critical values, which implies that there was no difference between predicted and observed data points. The training phase produced 99.99% difference between F statistic and F critical compared with validation (82.06%) and testing (97.23%) phases. This justifies the fact that the training and testing phases predicted the observed data accurately compared with the validation phase. Similarly, the *p* value was greater than 0.05, which indicates that there was no statistical difference between observed and predicted data.

**TABLE 5:** Observed versus predicted data ANOVA test.

Learning phase	F statistic	*p* value	F critical
Training	0.0000926	0.992	3.862
Validation	0.707	0.402	3.94
Testing	0.109	0.741	3.94

#### 3.3.2 MLP ANN model performance

**FIGURE 14:**
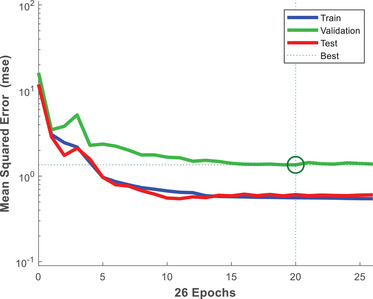
Relationship between MSE and number of iterations (Epochs).

The MLP ANN model performance is presented in Table 6. MSE during training, validation, and testing phases were 0.5589, 1.3551, and 0.6081, respectively. The optimum performance was determined during the training phase, which justifies the results of the prediction accuracy ANOVA test. This was because the training phase (228) had a larger dataset compared with the validation (48) and testing (48) phases. The relationship between MSE and number of iterations (Epochs) is shown in Figure 14. As shown in Figure 14, the lowest MSE (0.5589) was determined during the training phase. During the validation phase, the model struggled to predict the observed values hence, MSE of 1.3551 was determined. The training and testing phases value were close to zero, which indicates that the MLP ANN algorithm was successful in modeling biomass growth data in the biological treatment process.

**TABLE 6:** MLP ANN model performance

	Training	Validation	Testing
**MSE**	0.5589	1.3551	0.6081
**RMSE**	0.7476	1.1641	0.7798
***R* ^2^**	0.844	0.853	0.823

**FIGURE 15:**
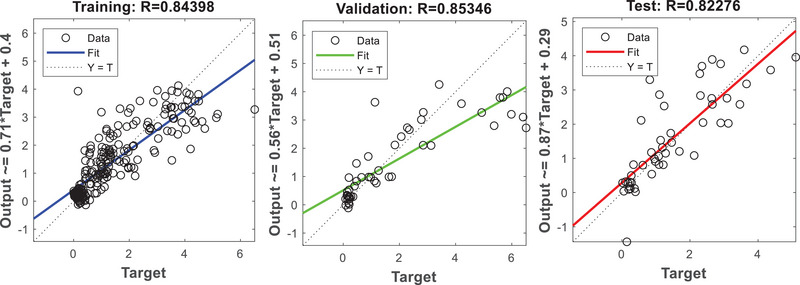
*R* ^2^ values during training (A), validation (B), and testing (C) phases.

Similarly, the optimum RMSE (0.7476) was determined during the training phase compared with the validation and testing phases. During the validation phase, RMSE was greater than one, which suggests that MLP ANN algorithm struggled to model the biomass growth data. However, the overall performance of the model was satisfactory because the MSE and RMSE values during the training and testing phases were close to zero. The *R* ^2^ values also justified that the MLP ANN algorithm performed well during the training (0.844), validation (0.853), and testing (0.823) phases of the model as shown in Figure 15. The *R* ^2^ values during training, validation, and testing phases were close to the value of one, which implies that the model will be able to predict the observed values accurately.

Table 7 presents ANN algorithm performance results obtained from other studies. The average reported *R* ^2^ value was 0.8914, which is similar to the current study results (0.823) as shown in Table 7. The difference between the average *R* ^2^ and current *R* ^2^ value was 7.7%, which was minimal. However, the average reported MSE of 0.428 was lower compared with current study results (0.6081). This is a total difference of 29.62% between the average MSE and the current study results, which is not significant. The average RMSE value was 16.212, which was higher compared with the results obtained in the current study (0.7798). This implies that the results obtained in the current study were superior compared with results obtained by different scholars. Therefore, the MLP ANN algorithm produced robust results.

**TABLE 7:** ANN algorithm performance comparison results

No	*R* ^2^	MSE	RMSE	REFERENCES
1	0.823	0.6081	0.7798	Current study
2	0.87	0.056	–	[51]
3	–	0.0041	–	[52]
4	–	2.81	–	[53]
5	0.81	–	29.69	[54]
6	0.85	–	3.23	[55]
7	0.67	–	–	[56]
8	0.885	–	–	[57]
9	0.97	–	–	[58]
10	0.98	–	–	[59]
11	0.99	$2.317\times 10^{-15}$	0.8673	[60]
12	–	–	0.101	[61]
13	0.895	0.0033	–	[62]
14	0.933	0.0175	–	[63]
15	0.754	–	–	[64]
16	0.99	–	–	[65]
17	0.961	–	–	[66]
18	–	0.468	0.6840	[67]
19	–	–	115.85	[68]
20	–	–	0.04	[69]
21	0.99	–	–	[70]
22	–	0.000441	–	[71]
23	–	0.311	–	[72]
24	–	–	6.95	[73]
25	–	–	3.93	[74]
**Highest**	**0.99**	**2.81**	**115.85**	
**Average**	**0.8914**	**0.428**	**16.212**	
**Lowest**	**0.67**		**0.04**	

#### 3.3.3 Sensitivity analysis

**FIGURE 16:**
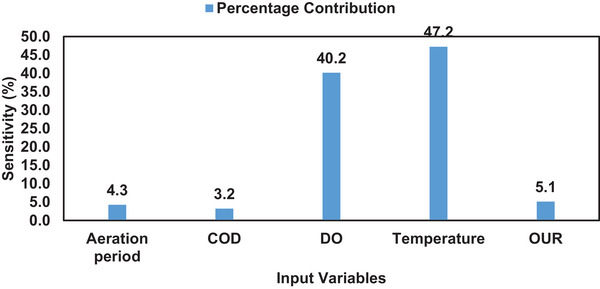
Sensitivity analysis on the biomass concentration model.

Sensitivity analysis was performed on the MLP ANN biomass growth model as shown in Figure 16. The results showed that temperature (47.2%) and DO concentration (40.2%) were the biggest drivers of biomass growth in the biological treatment process. High temperatures (32.5°C) have shown that they improve the metabolic rate of microorganisms, resulting in high biomass growth [35, 75, 76, 77]. This indicates that the biological treatment process should be operated at high temperatures (32.5°C) in order to improve the biomass growth. DO concentration is essential for the respiration of microorganisms hence it contributes highly toward the growth of microorganisms [78]. This implies that adequate DO concentration should be maintained in the biological treatment process.

Aeration period (4.3%) contributed minimally toward biomass growth as shown in Figure 16. This is evidence that longer/extended aeration periods do not guarantee biomass growth but rather consume high levels of energy, since additional airflow supply will be required [79]. This is due to the fact that growth of microorganisms depends on the availability of nutrients, and DO concentration, hence aeration period contributes minimally [80]. Without the availability of the parameters (nutrients and DO concentration), microorganisms will enter the stationary and death phases which result in a reduction of microorganisms. Therefore, extending aeration period will not yield significant biomass growth in the biological treatment process.

Similarly, COD concentration (3.2%) contributed minimally toward biomass growth in the biomass treatment process. This implies that substrate (COD) present in wastewater is not sufficient to ensure rapid biomass growth in the biological treatment process. Nutrients can be consumed and depleted without achieving maximum biomass growth hence, COD concentration contributed minimally. In addition, the presence of toxic substrate contributes toward inhibiting biomass growth [81, 82]. Lastly, OUR (5.1%) contributed minimally toward biomass growth in the biological treatment process. OUR represents the oxygen consumed by microorganisms in the biological treatment process. The oxygen consumption does not guarantee biomass growth but rather indicates the respiration of microorganisms.

**FIGURE 17:**
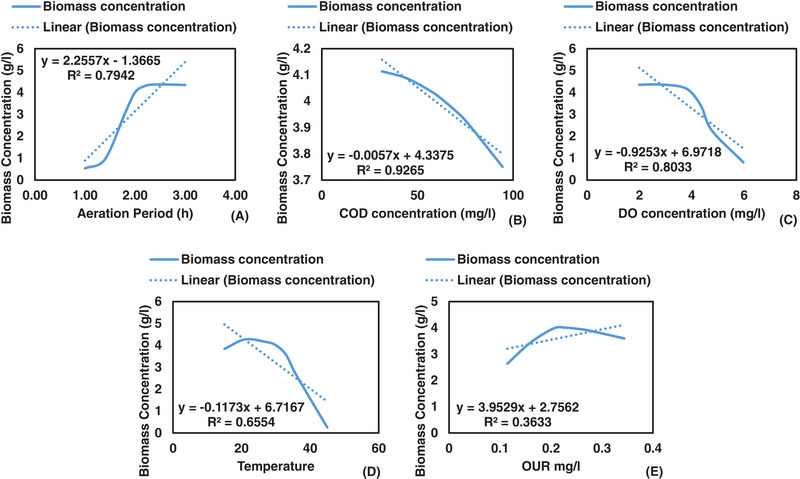
Effects of aeration period (A), COD (B), DO (C), temperature (D), and OUR (E) on biomass concentration.

The relationship between biomass growth model and input variables are presented in Figure 17A–E. The relationship between biomass growth model and aeration period showed an S shaped curved, with the peak growth reached at the two‐hour mark. Three growth phases (lag, exponential, stationary phases) can be observed in the biological treatment process between hour one and hour three as shown in Figure 17A. This indicates that microorganisms were still available in abundance between the first and third hour. It can also be observed that the stationary phase was reached at the 2‐hour mark, which indicates that nutrients were depleted at that point.

The relationship between biomass growth and aeration period showed a positive linear regression of 2.255 g/L and *R* ^2^ value of 0.794. Other scholars reported that microorganisms struggled to transition from the lag phase to the exponential phase, which was not the case in the results obtained in the current study [83, 84]. Biomass concentration increased with a decrease in COD concentration as shown in Figure 17B. This was expected since COD concentration is the nutrients that microorganisms feed on, hence when biomass increase COD concentration will decrease. The rate of change in biomass caused by the change in COD concentration was negative 0.0057 g/L. This was minimal which further justifies that the consumption of nutrients contributes a small margin toward biomass growth. Similar results were reported by other scholars [85].

Biomass concentration increased with a decrease in DO concentration in the biological treatment process as shown in Figure 17C. The rate of change in biomass growth caused by the change in DO concentration was negative 0.9253 g/L. This indicates that the rate of change in biomass influenced by the change in DO concentration was 0.9253 g/L. *R* ^2^ value of 0.8033 shows that 80.33% of the variation in biomass growth is explained by DO concentration. This is because microorganisms use DO concentration present in wastewater for respiration purposes, hence when microorganisms increase in population, the demand in DO concentration increases, resulting in a decrease of DO concentration. Similar results were reported by other scholars [86, 87].

Biomass growth reached the peak at a temperature of 30°C, beyond that temperature, biomass growth declined linearly as shown in Figure 17D. This implies that the maximum growth of microorganisms in the wastewater was 30°C. The group of microorganisms that are found in wastewater are classified as mesophilic, and grow in the temperature range between 10°C and 45°C, with an optimum growth of 32.5°C [88, 89]. Therefore, to ensure that microorganisms stay alive and maintain maximum growth, temperature should not exceed 30°C. Similar results have been reported by scholars [90, 91].

The peak OUR was measured at 0.226 mg/L, which indicates that the stationary and death phase have been reached hence, a decline in OUR took place beyond that point. The rate of change in biomass caused by the change in OUR was 3.9529 g/L, but the *R* ^2^ value (0.3633) showed that the data did not follow a linear trend. The stationary phase takes place due to the depletion of nutrients and the presence of toxic substrate in the biological treatment process. OUR is a useful parameter which can be used to monitor the health of microorganisms hence, it has been used to monitor the physiological state of microorganisms in the biological treatment process [92].

## 4 CONCLUDING REMARKS

The biological treatment process is essential for decomposing organic and oxidizing inorganic matter present in wastewater. The process is achieved using microorganisms that are present in the wastewater. The challenge experienced is that the growth of microorganisms is slow, which slows down the removal of organic and inorganic matter. The slow removal of organic and inorganic matter will require extended aeration period which consume high levels of energy. The aim of the research was to model biomass growth in attempts to improve the growth of microorganisms in the biological treatment process.

MLP ANN algorithm was used to model biomass growth in the biological treatment process. Laboratory experiments were used to collect input data that was used to model biomass growth. The model performance was measured using *R* ^2^, MSE, and RMSE. Sensitivity analysis was performed in order to evaluate variables that have strong influence on the biomass growth model. The MLP ANN algorithm modeled the biomass growth successfully and showed good prediction accuracy. *R* ^2^, MSE, and RMSE were 0.823, 0.7798, and 0.6081 during the testing phase respectively. Temperature and DO concentration showed that they are the biggest drivers of biomass growth in the biological treatment process.

This implies that in order to achieved maximum biomass growth, wastewater temperature should be increased in the biological treatment process. The study recommends that the biological treatment process should be operated at high wastewater temperature in order to achieve maximum growth of microorganisms. This will accelerate the removal of organic and inorganic matter in wastewater, resulting in an efficient and effective biological treatment process.

## Data Availability

Research data not shared.
